# Unveiling the molecular profile of adenosquamous gallbladder carcinoma: characterization of a Caucasian cohort

**DOI:** 10.1111/his.70179

**Published:** 2026-05-29

**Authors:** Jessica Gasparello, Valentina Angerilli, Mario Domenico Rizzato, Edoardo Maffii, Carlotta Ceccon, Matilde Callegarin, Marianna Sabbadin, Monia Niero, Luisa Toffolatti, Francesca Bergamo, Paola Parente, Luca Mastracci, Federica Grillo, Alessandro Vanoli, Paola Mattiolo, Claudio Luchini, Angelo Paolo Dei Tos, Umberto Cillo, Giacomo Zanus, Sara Lonardi, Matteo Fassan

**Affiliations:** ^1^ Department of Medicine University of Padua Padua Italy; ^2^ Surgical Pathology Unit ULSS2 Marca Trevigiana Treviso Italy; ^3^ Department of Pathology, Radboud Institute for Molecular Life Sciences Radboud University Medical Centre Nijmegen The Netherlands; ^4^ Veneto Institute of Oncology IOV‐IRCCS Padua Italy; ^5^ Pathology Unit Azienda Ospedaliero‐Universitaria Policlinico Riuniti Foggia Italy; ^6^ Pathology Unit, Department of Surgical Sciences and Integrated Diagnostics (DISC) University of Genoa Genoa Italy; ^7^ IRCCS Azienda Ospedaliera Metropolitana (IRCCS AOM) Ospedale Policlinico San Martino Genoa Italy; ^8^ Fondazione IRCCS Policlinico San Matteo Pavia Italy; ^9^ Department of Pathology and Diagnostics University and Hospital Trust of Verona Verona Italy; ^10^ Department of Surgical, Oncological and Gastroenterological Sciences (DISCOG) University of Padua Padua Italy; ^11^ Department of Surgery, General Surgery 2‐HPB Surgery and Liver Transplantation Unit Padua University Hospital Padua Italy; ^12^ General Surgery 2 ULSS2 Marca Trevigiana Treviso Italy

**Keywords:** adenosquamous gallbladder carcinoma, gallbladder cancer, molecular profile, next‐generation sequencing, targeted therapy

## Abstract

**Aims:**

Gallbladder carcinoma (GBC) is a rare and highly aggressive malignancy associated with poor clinical outcomes. While most GBCs are adenocarcinomas, only a small subset shows squamous differentiation. Tumours containing more than 25% squamous component are classified as gallbladder adenosquamous carcinomas (GBASC), a rare histological subtype characterized by a significantly worse prognosis than conventional adenocarcinomas. Due to their rarity, the molecular landscape and biological basis underlying the aggressive behaviour of GBASCs remain poorly understood.

**Methods and results:**

In this study, 25 retrospectively collected GBASC cases were comprehensively characterized at both immunohistochemical and molecular levels. Predictive biomarkers including programmed death‐ligand 1 (PD‐L1), mismatch repair proteins (MMR), human epidermal growth factor receptor 2 (HER2) and claudin 18.2 (CLDN18.2) were evaluated. Nearly all tumours (96%) showed PD‐L1 positivity with a combined positive score (CPS) greater than 1. CLDN18.2 expression was identified in 28% of cases, although its confinement to the glandular component may limit its therapeutic applicability. All tumours were mismatch repair proficient (MMRp), and none demonstrated HER2 overexpression, in contrast to a subset of gallbladder adenocarcinomas. Molecular profiling revealed recurrent alterations in *TP53* (64%), *KRAS* (16%) and *CDKN2A* (20%), partially overlapping with the genomic profile of gallbladder adenocarcinomas. However, GBASCs showed a significant enrichment of alterations involving the PI3K pathway, particularly *PIK3CA* (44%) and *PTEN* (24%) mutations, representing their most distinctive molecular feature.

**Conclusions:**

Overall, these findings support the recognition of GBASC as a distinct molecular entity potentially requiring dedicated therapeutic strategies.

## Graphical abstract

GBASCs are MMRp tumours, exhibiting minimal HER2 overexpression and elevated PD‐L1 expression compared to adenocarcinomas. Especially in metastatic and *TP53*‐wild type tumours, GBASCs can express CLDN18, albeit limited to the glandular component. Their genomic profile resembles that of adenocarcinomas with an enrichment in alterations on *PIK3CA*, *PTEN* and *HNF1A* genes.

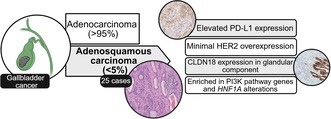

**Abbreviations**
BilIN: biliary intraepithelial neoplasia
BTC: biliary tract cancers
CLDN18: Claudin 18
CNV: copy number variation
CPS: combined positive score
FFPE: formalin fixed paraffin embedded
GBASC: adenosquamous gallbladder carcinoma
GBC: gallbladder carcinoma
H&E: haematoxylin and eosin
ICPN: intracholecystic papillary neoplasm
MMR: mismatch repair
MMRd: MMR deficient
SNV: single nucleotide variations
TPS: tumour proportion score

## Introduction

Gallbladder carcinoma (GBC) is a rare and fatal malignancy that represents nearly half of all cancers of the biliary tract.^1^, ^2^ Late diagnosis and a high rate of relapse after surgery (possible in only 10%–20% of patients) dramatically reduce the survival rate of patients with GBC, which does not exceed 10% at 5 years.^3^, ^4^ Adenocarcinoma is by far the most common subtype, representing more than 90% of all GBC, while adenosquamous gallbladder carcinoma (GBASC) has a significantly lower incidence, representing less than 5% of all GBC cases.^5^, ^6^ GBASCs are characterized by distinct glandular and squamous differentiation. According to the WHO diagnostic criteria for adenosquamous cancer, except for pancreatic adenosquamous carcinoma, the proportion of squamous component must be at least 25%.^7^ Generally, GBCs are characterized by aggressive biology with frequent extension to adjacent structures, including the liver, omentum, stomach, duodenum and transverse colon. In the few available studies, the squamous component often displayed a greater proliferative capacity, resulting in faster and more aggressive growth with the infiltration of adjacent organs and consequently worse prognosis.^8^

Regarding molecular pathology, GBCs are characterized by a high incidence (57% of all GBCs) of somatic single‐nucleotide variations (SNVs) in the *TP53* gene, which inactivate the p53 oncosuppressor protein.^9^, ^10^ Other common genetic alterations involve *SHH* (20%), *ELF3* (19%), *PIK3CA* (14.6%), *ARID1A* (14%), *SMAD4* (13%), *EGFR* (12%), *KRAS* (10.3%) and *ERBB2* (10%).^9^, ^11^, ^12^ Unfortunately, none of the molecularly evaluated series adequately distinguishes GBCs by histotype.

Due to their rarity, GBASC molecular profiles are mainly reported as case reports, and limited data are available. Galuppini *et al*., in a GBASC case, where glandular and squamous components coexisted but were distinguishable, investigated the mutational profile in 11 cancer‐driver genes, comparing the two micro‐dissected components.^13^ Both tumour components harboured the *PIK3CA* p.E542K point mutation; the adenocarcinoma component also presented additional mutations, such as the *TP53* p.Q331* stop mutation, which was not detected in the squamous component. Thus, different histotypes may present different molecular landscapes, partially explaining different clinical behaviour. In the present work, the molecular profile of one of the most numerous Caucasian GBASC cohorts has been evaluated using an extended NGS panel.

## Materials and Methods

### Case Selection

The investigational cohort consisted of 25 patients who received the diagnosis of GBASC, and underwent surgery between 2007 and 2022. Cases, all surgical specimens, were obtained from different Italian institutions, including: ULSS2 General Hospital (Treviso), Padua University Hospital (Padua, Italy), University and Hospital Trust of Verona, Ospedale Policlinico San Martino IRCCS (Genoa, Italy), Fondazione IRCCS Casa Sollievo della Sofferenza (San Giovanni Rotondo, Italy) and Fondazione IRCCS Policlinico San Matteo (Pavia, Italy).

Haematoxylin and eosin (H&E) stained sections were blinded and reviewed by two expert gastrointestinal pathologists (MF and VA), and according to the WHO diagnostic criteria, only cases presenting at least 25% of the squamous component were selected. H&E slides were also evaluated to establish the TNM stage and tumour grade, according to the 6th edition of the WHO Digestive Tumours Classification, the presence and grade of premalignant lesions (e.g. BilIN or ICPN), and the presence of intestinal metaplasia.

All clinical and demographic information about human tissue was managed using anonymous alphanumeric codes, and all samples were handled according to the Declaration of Helsinki. All cases were collected according to the BITCOIN protocol, approved by the local Ethic Committee (EM 2024‐34, 05/15/2024).

### Immunohistochemical Analysis

Stains were performed using the Bond Polymer Refine Detection Kit (Leica Biosystems, Deer Park, IL, USA) on the BOND‐MAX automated IHC stainer (Leica Biosystems). Three μm‐thick formalin‐fixed paraffin‐embedded (FFPE) sections were incubated with the following primary antibodies according to the manufacturer's instructions. (a, b) p40 (clone BC28, Leica Biosystem) and p63 (clone BC4A4; Bio Optica, Milan, Italy) stains were performed to confirm neoplastic areas presenting squamous histology. (c) HER2 (clone 4B5, Roche‐Diagnostics‐Ventana, Basel, Switzerland) expression was evaluated according to the 4‐tiered Hoffmann scoring criteria.^14^ In the presence of a complete or basolateral membranous reactivity in ≥10% of tumour cells, scores of 1+ (faint), 2+ (moderate) and 3+ (intense) were assigned according to the intensity of membranous reactivity. (d) Mismatch repair (MMR) status was assessed by testing MLH1 (clone ES05; Dako‐Agilent Technologies, Santa Clara, CA, USA), PMS2 (clone EP51; Dako‐Agilent Technologies), MSH2 (clone 79H11; Dako‐Agilent Technologies) and MSH6 (clone EP49; Dako‐Agilent Technologies) proteins. Samples were defined as MMR‐deficient (MMRd) when one or both of the proteins constituting the heterodimer were not expressed in the presence of an adequate internal positive control (intratumour stromal cells and non‐neoplastic cells).^15^ (e) PD‐L1 (clone 22C3, Dako‐Agilent Technologies) expression was calculated according to the combined positive score (CPS) and tumour proportion score (TPS).^16^ (f) Claudin 18 (CLDN18) immunoreactivity (clone 43‐14A; Roche Diagnostics‐Ventana) was evaluated, reporting the percentage of stained tumour cells. Tumour was defined as positive for CLDN18 expression when more than 75% of tumour cells presented a 2 + / 3 + intensity score.^17^

### Targeted Next‐Generation Sequencing

For each sample, two experienced pathologists (MF and VA) evaluated the H&E slices and carefully marked the representative neoplastic area, indicating the percentage of neoplastic cells. Five consecutive 5‐μm‐thick sections were obtained from each FFPE block, and the previously marked areas were manually macro‐dissected, including in the same sample both the glandular and the squamous components.

Macro‐dissected tissues were dewaxed using heat and a deparaffinization solution (Qiagen, Hilden, Germany). DNA was then manually extracted using the QIAmp FFPE tissue kit (Qiagen), according to the manufacturer's instructions. The concentration of the obtained DNA samples was evaluated using the Qubit 4.0 fluorometer and the respective Qubit high‐sensitivity DNA assay kit (Thermo Fisher Scientific, Waltham, MA, USA). The quality of the extracted DNA was assessed using the Archer PreSeq DNA QC assay (Archer Integrated DNA Technology, IDT; Coralville, Iowa, USA), and the QC Score was calculated. QC Score indicates the fragmentation of each sample and was used to determine the number of amplifiable genomes per ng of DNA.

Libraries were prepared using Archer VariantPlex solid tumour panel (Archer Integrated DNA Technology), which was able to analyse 660 regions of interest across 67 frequently mutated genes in a broad spectrum of solid tumours. The complete list of investigated genes has been reported in Table S1. The assay that uses anchored multiplex technology to enrich regions of interest can detect single‐nucleotide variants (SNV), indels (63 genes) and copy number variation (CNV, 44 genes). For each sample, 250 ng of total DNA were used to create libraries according to the manufacturer's protocol. After quantification, the size of each library was calculated using TapeStation 4200 (Agilent Technologies) and the respective D1000 high‐sensitivity reagents (Agilent Technologies).

The libraries were pooled at an equimolar concentration. The obtained final pool was denatured using a 0.2 N NaOH solution, and subsequently diluted to a final concentration of 1.6 pM. Libraries were sequenced on the NextSeq‐550Dx platform using the NextSeq 500/550 mid‐output kit V2.5 (Illumina, San Diego, CA, USA). Sequenced fragments were converted into demultiplexed FASTQ files and processed using Archer Analysis software (v.7.3; Archer‐Integrated DNA Technology). Manufacturer quality control criteria were used to determine whether the results were valid, and SNVs were classified according to the TIER classification by searching for mutations on three different databases: Franklin‐Genoox (https://franklin.genoox.com/clinical‐db/home), ClinVar (https://www.ncbi.nlm.nih.gov/clinvar/) and cBioPortal (https://www.cbioportal.org/).

### Statistics

Differences between categorical and continuous variables were assessed respectively by two‐tailed *χ* ^2^ test and Student's *t*‐test or ANOVA test, when more than two categories were tested. Statistical analyses were carried out using GraphPad Prism version 5 (Boston, MA, USA). *p* <0.05 were considered statistically significant.

## Results

### Clinicopathological Data

**Figure 1:**
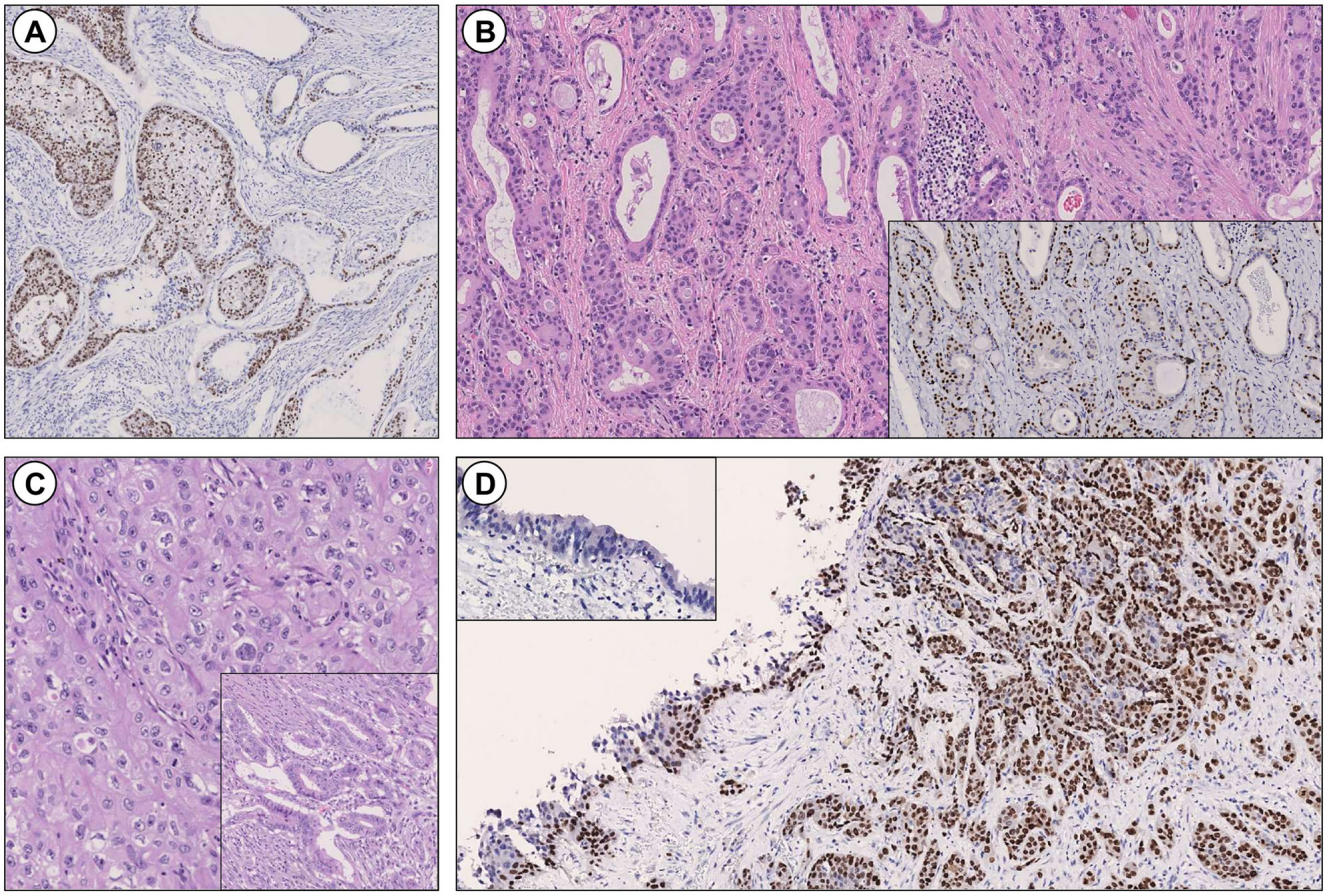
Representative images of gallbladder adenosquamous carcinomas. H&E and/or p63 staining are shown. (**A**) Transition between glandular and squamous component (p63, IHC). (**B**) Intermingled glandular and squamous components (H&E; inlet: p63 IHC). (**C**) Squamous component at higher magnification (H&E; inlet: glandular component); (**D**) Gallbladder adenosquamous carcinoma arising from high‐grade biliary intraepithelial neoplasia (p63 IHC; inlet low‐grade biliary intraepithelial neoplasia).

The investigated cohort was composed of 25 patients: 13 were female (52%) and 12 were male (48%), and the median age at diagnosis was 69.5 years (range 53–83). No significant differences in gender (*P =* 0.8415) or median age between males and females (68.8 years for the males, 67 years for the females; *P* = 0.3106) were reported. Most of the patients (23/25; 92%) presented at diagnosis with locally advanced disease (T3 or T4), while only two patients (8%) presented with a malignancy limited to the gallbladder. Nine patients (36%) presented lymph node metastases at diagnosis (8/25 staged N1 and 1/25 staged N2), and seven patients (28%) presented distant metastases. The squamous component in the investigated cohort ranged from 30% to 95% (Figure 1).

Twenty patients (80%) presented biliary intraepithelial neoplasia (BilIN), in all cases, high‐grade (HG‐BilIN) lesions were observed. In five cases (20%), the concomitant presence of HG‐BilIN and intestinal metaplasia was reported. In one case (4%), both high‐grade intracholecystic papillary neoplasm (HG‐ICPN) and HG‐BilIN lesions were identified. One case (GBC‐2) presented GBASC with sarcomatoid features of the squamous component, while another case (GBC‐6) presented focal neuroendocrine differentiation (with synaptophysin expression in 10% of the glandular component). In one case (GBC‐21), an area with poorly cohesive signet ring‐type cells was observed. Clinico‐pathological and phenotypical features of the analysed cases are summarized in Table 1.

**Table 1:** Clinicopathological and immunohistochemical features of the investigated cohort

Clinicopathological and immunohistochemical features of the investigated cohort					
Sex	Male	12/25 (48%)	Age at diagnosis	<65	8/25 (32%)
	Female	13/25 (52%)		>65	17/25 (68%)
pT	T1	1/25 (4%)	pN	N0	10/25 (40%)
	T2	1/25 (4%)		N1	8/25 (32%)
	T3	19/25 (76%)		N2	1/25 (4%)
	T4	4/25 (16%)		Nx	6/25 (24%)
pM	M0	18/25 (72%)	Squamous component	<75%	14/25 (56%)
	M1	7/25 (28%)		≥75%	11/25 (44%)
BilIN	Absent	5/25 (20%)	Intestinal metaplasia	Yes	5/25 (20%)
	Present	20/25 (80%)		No	20/25 (80%)
p63	Positive	24/25 (96%)	p40	Positive	23/25 (92%)
	Negative	1/25 (4%)		Negative	2/25 (8%)
MMR proteins	MMRp	25/25 (100%)	CLDN18*	Positive	7/25 (28%)
	MMRd	0/25 (0%)		Negative	18/25 (72%)
PD‐L1 (CPS score)	<1	0 (%)	PD‐L1 (TPS score %)	<1	17/25 (68%)
	1 ≤ CPS < 10	7/25 (28%)		1 < TPS < 10	2/25 (8%)
	10 ≤ CPS < 50	11/25 (44%)		10 < TPS < 50	4/25 (16%)
	≥50	6/25 (24%)		>50	1/25 (4%)
	NV	1/25 (4%)		NV	1/25 (4%)
HER2	0	22/25 (88%)	HER2	2+	0/25 (0%)
	1+	3/25 (12%)		3+	0/25 (0%)

BilIN, biliary intraepithelial neoplasia; claudin‐18; CLDN18; CPS, Combined Positive Score; HG, high grade; LG, low grade; MMR, mismatch repair; NV, not evaluable; TPS, Tumour Proportion Score.

* Evaluated on the glandular component.

### Immunohistochemical Characterization of GBASCs

Immunohistochemical determinations to confirm the squamous differentiation were performed by evaluating the nuclear expression of p63 and p40 proteins. All 25 cases, except for GBC‐8 (96%) were positive for p63 staining, and 23 cases (92%) were positive for p40. Both p40‐negative cases were positive for p63, while GBC‐8 was found to be negative for p63 but positive for p40. The squamous component was intermingled with the glandular component in 14 cases (56%) and was distinct from the glandular component in 11 cases (44%).

None of the investigated cases exhibited HER2 overexpression. According to the Hoffman criteria, a weak positivity in the glandular component (score 1+) was reported for three cases (12%).

PD‐L1 expression was assessed in the whole cohort using both CPS and TPS scores. One case (GBC‐8) was deemed inadequate for IHC evaluation. All the assessed cases presented a CPS score higher than 1, applying more stringent cut‐off, such as >10 and >50, respectively, 18 (72%) and 6 (24%) cases were positive for the PD‐L1 immunoreaction. TPS values were notably lower, with only eight cases (32%) displaying PD‐L1‐positive tumour cells. Of these, five cases (20%) presented with a TPS >10%, and only one case (GBC‐2) exhibited a TPS >50%. Furthermore, all the investigated cases presented a proficient MMR (MMRp) profile. A summary of the investigated IHC markers is reported in Table 1.

**Figure 2:**
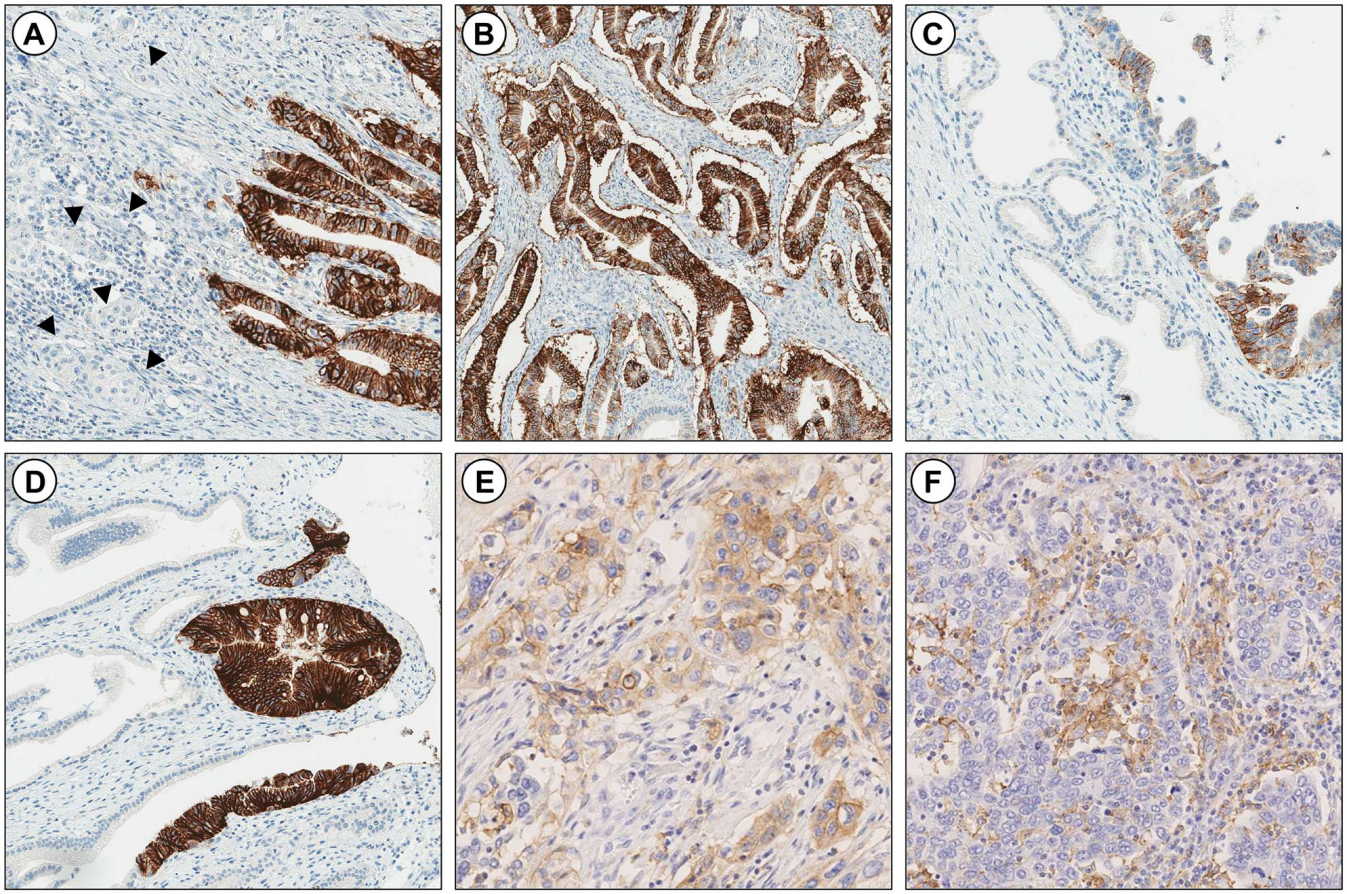
Representative IHC staining. (**A**) Claudin18.2‐positive glandular component adjacent to Claudin18.2‐negative squamous component (black arrows); (**B**) Claudin18.2‐positive glandular component of gallbladder adenosquamous carcinoma; (**C, D**) Biliary intraepithelial neoplasia showing claudin18.2 expression; (**E**) Representative case of gallbladder adenosquamous carcinoma with high PD‐L1 expression within tumour cells in the squamous component and (**F**) high PD‐L1 expression within inflammatory cells in the glandular component.

According to recent findings regarding Claudin 18.2 (CLDN18.2) expression in biliary tract cancers, including GBCs, CLDN18.2 was assessed in all cohort cases. Using a cut‐off value previously reported by Angerilli *et al*., and currently employed in gastric cancer clinical practice, 7 out of 25 cases (28%) were found to be positive for CLDN18 expression.^18^ In all cases, positivity was observed exclusively in the glandular component, with multifocal positivity seen in the BilIN lesions (Figure 2). Interestingly, of the seven CLDN18.2‐positive cases, four (57.1%) were metastatic, with a significant correlation (*p = 0.0430*) between CLDN18.2 positivity and metastatic disease (Table S2). Moreover, while the vast majority (87.5%) of *TP53*‐mutated cases were negative for CLDN18 expression, five out of the nine wild‐type cases (55.6%) expressed CLDN18, indicating a possible negative correlation between *TP53* mutations and CLDN‐18 expression (*P =* 0.0214).

### Molecular Characterization of GBASCs

According to the quality check (QC) performed before the library preparation, two samples (GBC‐8 and GBC‐16) presented highly fragmented DNA. Nevertheless, libraries were prepared and sequenced from both samples. At post‐sequencing QC, GBC‐16 presented with good metrics, in line with those reported for the other samples, while GBC‐8 showed suboptimal, despite acceptable, coverage metrics.

**Figure 3:**
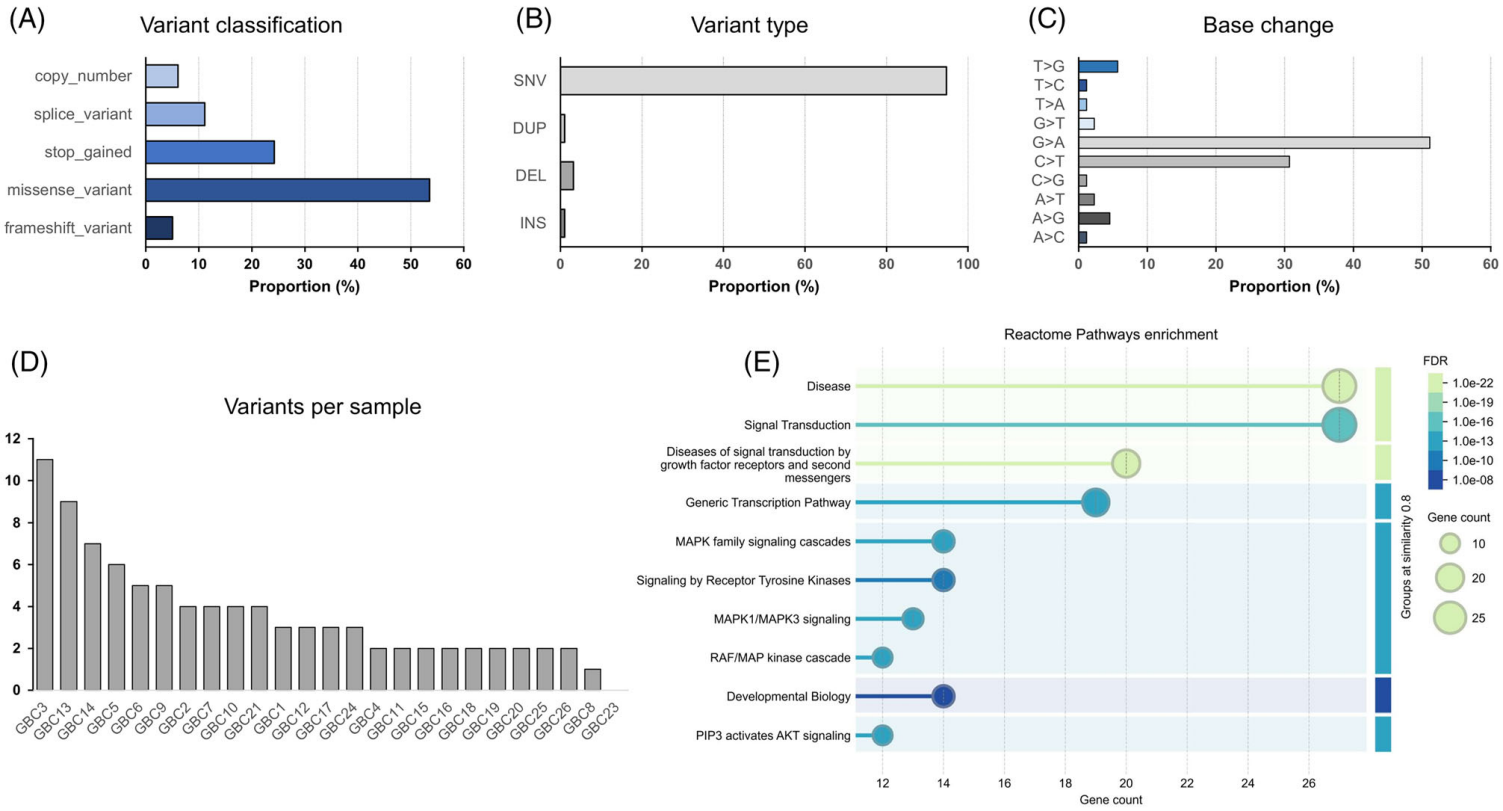
Principal features of the genetic alterations detected in the analysed cohort. (**A**–**C**) The distribution of principal genetic alterations is summarized. (**D**) The number of genetic alterations identified for each sample is summarized in the plot. (**E**) The figure provides a report on the molecular pathways in which mutated genes are involved, according to the Reactome Pathway Database. DEL, deletion; DUP: duplication; INS, insertion; SNV, single‐nucleotide variation. [Colour figure can be viewed at wileyonlinelibrary.com]

All detected alterations, including SNV, indels, splice‐site alterations (allele frequency: AF; cut‐off: 3%) and CNVs, are reported in Figure 3. In 24 out of 25 selected patients (96%), at least one genetic alteration was reported. Only one patient did not present any pathogenic alteration in the 67 investigated genes. The number of detected genetic alterations per patient ranged from 0 to 11, with a median of three genetic alterations per patient. Four patients, GBC‐3 (11 alterations), GBC‐5 (6 alterations), GBC‐13 (9 alterations) and GBC‐14 (7 alterations) presented at least six alterations. GBC‐3 presented the highest number of mutated genes: 11, with AFs ranging from 3.7% to 22.4%, and *PIK3CA* (AF: 22.4%) and *TP53* (AF: 13%) standing out as the most represented mutations. GBC‐13 presented nine mutated genes with homogeneous AFs, among the mutated genes (AF range 3.10–9.50%), while GBC‐14 (7 mutated genes) presented a more heterogeneous AFs profile (3.45%–39.67%), with *CDKN2A* mutation (AF: 39.67%) as the prevalent alteration. Twenty‐one out of 25 (84%) analysed cases had at least 1 SNV, 4/25 (16%) had at least 1 CNV and 1/25 (4%) had only a splice alteration.

Cases were classified according to the number of detected mutations/sample into three tiers: (i) less than 3, (ii) from 3 to 6 mutations/sample and (iii) more than 6 mutations/sample. The median value of the squamous component was 50% in the <3 subgroup, while a significantly higher percentage of squamous cells (median value 80%) was detected for the other two tiers: 3–6 mutations/sample and >6 mutations/sample (*P =* 0.0423). Furthermore, for all three cases presenting more than 6 mutations/sample, a percentage of squamous cells ≥75% was detected (*P =* 0.0331) (Table S3).

In the investigated cohort, the CPS score was associated with the number of mutations identified by NGS analysis. While for cases with CPS < 10, a mean of two mutations per sample was reported (SD ±0.38), 4.4 mutations (SD ±0.63) were detected with CPS > 10 (*P* = 0.0277).

**Figure 4:**
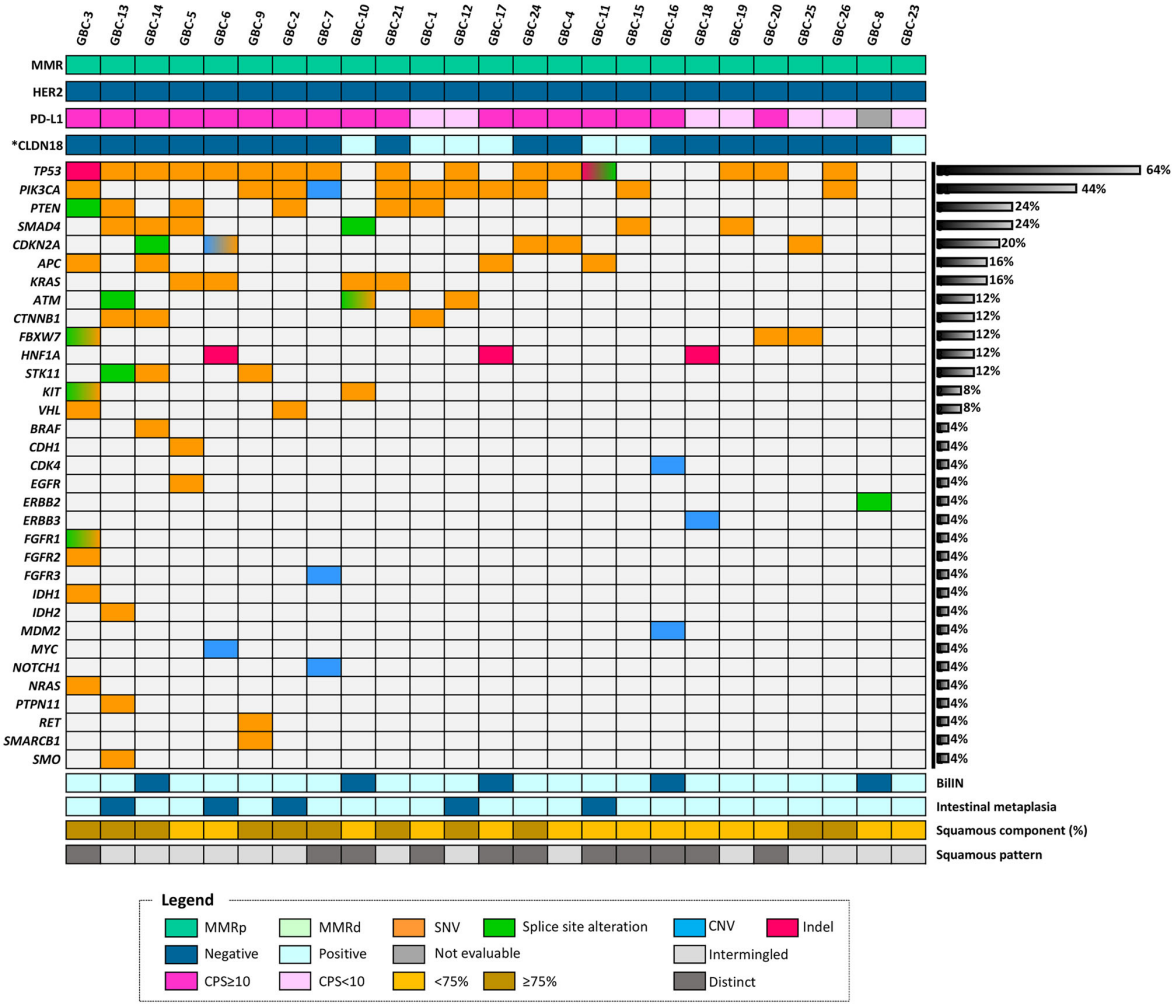
Oncoplot summarizing principal genetic alterations detected in the GBASC cohort. The percentage of cases presenting alterations for each gene is reported in the plot on the right. BilIN, Biliary intraepithelial neoplasia; CNV, copy number variation; indel, insertion/deletion; MMRd, mismatch repair deficient; MMRp, mismatch repair proficient; SNV, single‐nucleotide variant. *Positivity was evaluated on the glandular component.

The tumour suppressor *TP53* is the most frequently altered gene: 16 of the 25 patients (64%) presented alterations in this gene. In most cases (14/16, 88%) SNVs were detected; of these, two (14.3%) were nonsense mutations, while 12/14 (85.7%) were missense mutations (Figures 3 and 4). Only two cases were reported with alterations involving the splicing site, and one presented a base insertion. In two cases (GBC‐11 and GBC‐13), multiple *TP53* gene mutations were described. In GBC‐11, a splice donor alteration (AF: 18.1%) concomitant with a base insertion resulting in a frameshift change (AF: 4.5%) was detected, while GBC‐13 presented two SNVs, missense mutations with similar AF (p.Met237Ile 3.8% and p.Cys141Tyr 3.2%). Evaluating the mutation sites, two patients, GBC‐5 and GBC‐6, presented both mutations at the glycine (Gly) position 244, despite having different amino acid substitutions: p.Gly244Asp and p.Gly244Ser, respectively. Meanwhile, GBC‐4 and GBC‐20 showed missense mutations at the proline (Pro) position 278, resulting in p.Pro278Leu and p.Pro278Ser, amino acid substitutions. *TP53*‐mutated status can be associated with the presence of BilIN lesions (*P* = 0.0219). Moreover, a higher percentage of squamous component was detected in *TP53*‐mutated tumours compared with the *TP53*‐WT cases, with a median squamous component of 77.5% versus 50%, respectively. Although this difference is not significant, eight out of the nine wild‐type cases presented a squamous component lower than 75%.

Overall, *PIK3CA* alterations were reported in 11 out 25 investigated patients (prevalence 44%), 10 of them were point mutations resulting in missense mutations, while one patient presented *PIK3CA* gene amplification. Most frequently mutated amino acids were p.Glu545 (2/3: p.Glu545Lys; 1/3: p.Glu545Ala) and p.His1047 (2/3: p.His1047Arg; 1/3: p.His1047Leu), both mutated in 27.3% (3/11) of patients reporting *PIK3CA* SNV, followed by p.Glu542 (2/11; 18.2%; both p.Glu542Lys). Mutations on *PTEN*, part of the same molecular pathway as the *PIK3CA* gene, were found in 24% (6/25) of patients; of these 67% (4/6) presented concomitant alterations in both the *PTEN‐PIK3CA* axis. In all cases, except for GBC‐1, where similar AF were reported for both genes (4.3% vs 6%), *PIK3CA* mutation shows a sharp higher AF compared to *PTEN* (GBC‐2: 18% vs 4%; GBC‐3: 22.4% vs 3.4%; GBC‐21: 18.8% vs 3.9%). One out of the six altered cases resulted in a splicing mechanism alteration, while of the other five cases, three were nonsense point mutations and two were missense.

Similarly to *PTEN*, mutations in *SMAD4* and *CDKN2A* were observed in 24% and 20% of patients, respectively, with the p.Leu16Arg missense mutation reported in more than half (60%) of *CDKN2A‐*mutated patients. The two additional cases harboured splicing site alteration and a nonsense point mutation. In only one case (GBC‐6), *CDKN2A* copy number alteration was reported, and the loss of *CDKN2A* was concomitant with a missense mutation in the same gene. As regards *SMAD4* alterations: nonsense mutations (3/6), missense (3/6) and a splice site alteration (1/6) were detected, with one case (GBC‐13) presenting simultaneously a nonsense (p.Arg135Ter) and a missense (p.Asp493Asn) SNV, with similar AF (4.21% vs. 5.53%). All patients reporting mutations on *APC* (4/25; 16%) presented nonsense mutations causing APC protein truncation at the basic domain. In one of the *APC*‐mutated patients (25%), *APC* mutation was reported to be concomitant to *CTNNB1* mutation, which resulted altered in 12% (3/25) of cases investigated in the cohort. 16% of patients presented *KRAS* missense mutations; of them, two (GBC‐21 and GBC‐6) reported point mutations at codon 12 (p.Gly12Cys and p.Gly12Asp). In three out of four cases, the *KRAS* mutation was concurrent with *TP53* ones.

As regards the other genes of the *RAS* family, *NRAS* mutations were identified in one case (GBC‐3; missense mutation p.Val14Ile), while no *HRAS* mutations were detected. Despite at lower frequencies, mutations on *ATM* (12%, all nonsense mutations; AF: 5%–10%), *FBXW7 (*12%), *HNF1A (*12%; all frameshift alterations*), STK11* (12%; AF: 5.9–10%), *KIT* (8%) *and VHL* (8%) were found. Alterations on the *FGFR2* and *IDH1* genes were identified with a frequency of 4% for both. The point mutation p.Asp374Asn was reported in the *FGFR2* gene, while the druggable p.Arg132His was identified in *the IDH1* gene. Alterations on the number of gene copies were detected in four patients: GBC‐6 (*MYC* amplification associated with the loss of *CDKN2A*), GBC‐7 (amplification of *PIK3CA* and loss of *NOTCH1* and *FGFR3*), GBC‐16 (*MDM2* and *CDK4* amplification) and GBC‐18 (*ERBB3* amplification).

## Discussion

GBC is considered a rare entity, and GBASC, which represents <5% of all GBCs, is even less frequent.^19^ Data describing the genotype and phenotype of GBASC are almost anecdotal and limited to case reports describing one or a few cases.^20^, ^21^ This study aimed to address the existing knowledge gap by recruiting one of the largest European cohorts of GBASC, composed entirely of Caucasian patients. All patients were recruited from the same geographical region, which adds value to the study, given that the pathogenesis of GBC can be significantly influenced by environmental factors and ethnicity.^22^, ^23^, ^24^

The cohort investigated in this study was characterized from a histological, immunohistochemical and molecular point of view, allowing for the identification of both similarities and differences compared to the better‐characterized conventional gallbladder adenocarcinomas. In contrast to the cohorts predominantly comprising adenocarcinomas, which exhibit a female predominance, the cohort under investigation in this study demonstrated a more balanced sex distribution.^25^, ^26^, ^27^ This indicated a first important epidemiological difference that characterized GBASC, and which has already been underlined by previous studies.^28^ As would be expected, all but two of the investigated cases presented with locally advanced disease, and in one third of the cases with a metastatic malignancy.^8^, ^29^ This is consistent with the biological behaviour of squamous neoplasms, which have been shown to exhibit a growth rate that is twice that of adenocarcinomas.^30^

The presence of the squamous component, identified by histological evaluation, was also confirmed by the immunohistochemical detection of two squamous differentiation markers: p63 and p40.^31^ The data obtained in the gallbladder setting are consistent with those previously obtained in other tissues, such as the lung, where p63 was found to be more sensitive than p40, albeit with reduced specificity.^32^, ^33^ The combination of the two squamous markers is the best approach to confirm the squamous component (except when the tissue is scarce), especially for those cases presenting suboptimal pre‐analytics. Nevertheless, some cases could potentially not express either p63 or p40. Indeed, for example, in colorectal adenosquamous carcinoma, it was reported that only a fourth of the cases were positive for p63, while the positivity rate was even lower (13%) for p40.^34^, ^35^

To obtain a picture of possible druggable alterations in GBASC, immunohistochemical determination of some of the currently druggable markers was performed. Although with a certain degree of heterogeneity due to different cut‐offs and ethnicities, GBCs were shown to have the highest percentage of HER2 positivity among all BTCs, reaching 10%–20% of positive cases.^36^, ^37^, ^38^ Nevertheless, very limited data are available about HER2 expression in the adenosquamous histotype. De Bitter and colleagues, in their comprehensive characterization of a GBC cohort, included seven cases presenting with squamous/adenosquamous histotype. Of them, two cases were not tested for HER2 expression, while two cases presented with HER2 positivity, one with a 3+ score (prevalence 20%) and the other with a 1+ score.^9^ However, the evaluation was performed on tissue microarrays, which present some limitations in highlighting heterogeneity. To the best of our knowledge, our cohort represents one of the most extensive case histories of GBASC in which HER2 expression was tested. Except for two cases categorized as ‘HER2‐low’, the investigated GBASC cohort presented a markedly lower percentage of HER2‐positive cases (data also sustained by the molecular evaluation of *ERBB2* copies) than the gallbladder adenocarcinoma series.^39^

All the investigated cases presented a CPS≥1 (established as positivity cut‐off in the KEYNOTE‐966 trial), and 32% of cases presented a TPS score >1%.^40^, ^41^ Comparison with PD‐L1 expression data from adenocarcinoma cohorts suggests a higher, although not significant, PD‐L1 expression in the adenosquamous histotype, consistent with findings reported in previous studies.^42^, ^43^ Nevertheless, dedicated studies comparing adenocarcinomas and adenosquamous carcinomas are required to avoid biases (i.e. IHC platforms and protocols, inter‐observer variability).^16^ In our GBASC cohort, PD‐L1 expression was found to be related to the number of identified mutations (*P* = 0.0277), suggesting that a higher number of mutations is able to increase the neoantigenic load, resulting in PD1/PD‐L1 axis activation. This data should be further explored using more complex molecular panels able to provide data about tumour mutation burden (TMB) and the number of mutations per megabase (mut/Mb).

Finally, Claudin‐18.2 is emerging as a novel biomarker also in gallbladder cancer. Angerilli and colleagues reported that 15.6% of gallbladder adenocarcinomas were positive for CLDN18 expression (≥75% CLDN18‐positive neoplastic cells).^18^, ^44^, ^45^, ^46^ In our GBASC cohort, CLDN18 positivity was reported only in the glandular component of the tumour, and none of the cases presented CLDN18‐positivity in the squamous component, as already described in the gastric cancer setting, where some CLDN18‐positive adenosquamous carcinomas were tested.^47^ So, in GBASC, CLDN18 expression is expected to present (a) an intrinsic heterogeneity, due to the substantial negativity of the squamous component, and (b) a strong correlation with the percentage of the squamous component, which, by definition, is never lower than 25%. Therefore, this first overview suggested that CLDN18 testing in GBASC may be considered only in cases with a low squamous component. Nevertheless, the expression of CLDN18 in the glandular component was found to be associated with two distinct factors: firstly, the presence of metastatic disease (P = 0.0430) and secondly, the absence of *TP53* mutations (*P* = 0.0214). The correlation with advanced disease was not entirely unexpected, given that Kinzler *et al*. had previously reported a correlation between CLDN18 positivity and the presence of lymph nodes and distal metastasis, as well as perineural invasion, in a cohort of exclusively cholangiocarcinomas.^48^

The genomic characterization of GBASC revealed that 96% of GBASC presented at least one mutation, despite only a small fraction of them being currently actionable. A high number of mutations is correlated with a more abundant squamous component. This prompts the hypothesis that an elevated squamous component is associated with a greater number of genomic mutations, which, in turn, are linked to a higher neoantigenic load and immune ‘hot’ tumours characterized by increased PD‐L1 expression, in comparison with adenocarcinomas.

The percentage of *TP53*‐mutated cases is fully in agreement with meta‐analyses performed on gallbladder adenocarcinomas.^49^ Almost all cases harbouring *TP53* mutations presented BilIN lesions, suggesting a correlation between the presence of precursor lesions and *TP53* mutations (*P* = 0.0219) and a possible involvement of p53 protein since the first steps of gallbladder carcinogenesis. Further studies, in which only the BilIN component is micro‐dissected and sequenced, are required to confirm the presence of *TP53* mutations before the transformation of the precursor lesion into an invasive neoplasm and to enforce p53's role as a driver of gallbladder carcinogenesis. If *TP53* alterations are in line with those reported for adenocarcinomas, *PIK3CA* alterations were significantly more frequent in GBASC (Supplementary Figure S1). Most of the detected point mutations are located in exons 9 (p.E542K, p.E545A, p.E545K) and 20 (p.H1047R, p.H1047L), which are routinely tested in other neoplasms, such as metastatic breast cancer (ESCAT level of evidence: IA), where they are considered druggable, using the molecular targeting drug alpelisib.^50^, ^51^, ^52^, ^53^ Moreover, in 67% of cases, *PIK3CA* mutations were associated with *PTEN* mutations, which are part of the same molecular pathway. Even in this case, a sharply higher *PTEN*‐mutation rate was detected than in adenocarcinomas.^9^, ^10^, ^54^ Thus, we can speculate that alterations in the PIK3CA‐PTEN axis are a specific molecular fingerprint of the adenosquamous histotype.

Of relevance, the alteration of *hepatocyte nuclear factor 1A* (*HNF1A*), in our cohort, was reported in 12% of analysed cases. The same genetic variant was already described in microsatellite instability‐high colorectal cancer.^55^ As regards GBC, sporadic GBC series have reported *HNF1A* alterations.^56^, ^57^, ^58^ A preliminary association between *HNF1A* and dysregulated metabolic processes was suggested in the gallbladder, and further mechanistic studies will be necessary.^59^

Finally, one case was reported with the p.Arg132His *IDH1* mutation, which is more prevalent in intrahepatic cholangiocarcinoma; nevertheless, despite being rare, this point mutation has already been described in gallbladder adenocarcinomas, where the prevalence was reported of 1.1%, a bit lower than that described in our GBASC cohort.^60^

## Conclusions

In conclusion, GBASC, despite sharing some genotypic and phenotypic features with the better‐characterized adenocarcinoma, should be considered as a specific histological and molecular entity. A preliminary investigation has revealed that GBASC is predominantly an MMRp tumour, exhibiting minimal HER2 overexpression and elevated PD‐L1 expression in comparison to adenocarcinomas. Especially in metastatic settings and *TP53*‐wild type tumours, GBASCs can express CLDN18, albeit limited to the glandular component, thereby excluding GBASC from CLDN18‐targeting therapy. Molecularly, the genomic profile of GBASCs closely resembles that of adenocarcinomas with overlapping prevalence of driver mutations such as *TP53* and *KRAS*; however, it is enriched in alterations on genes involved in the PI3K pathway (*PIK3CA* and *PTEN*) and *HNF1A*. These findings raise two significant issues: (i) in GBC clinical practice, the design of the therapeutic protocol for GBC must take into account the different genotypes and phenotypes, (ii) the need for the correct identification of the squamous component, also with the aid of ancillary tests, such as p63 IHC determination. In addition, clinicians must be provided with a pathology report that clearly indicates the presence of the adenosquamous histotype.

## Author contributions

VA, JG and MF performed study concept and design, JG, VA, AV, FG and PP performed development of methodology and writing, LM, CL, PM, FB, SL, UC, APDT, LT, GM and MF reviewed and revised the manuscript; JG, VA, MN, MDR, EM, MC, CC and MS provided acquisition, analysis and interpretation of data, and statistical analysis; MN, MS, MC and CC provided technical and material support. All authors read and approved the final paper.

## Funding information

This work has been funded by the European Union (EU)‐Next‐Generation EU (‘PNRR M4C2‐Investimento 1.4‐CN00000041’).

## Conflict of interest statement

MF declares receiving research funding from Roche, Astellas and Diaceutics; receiving payment/honoraria from Roche, Astellas, AstraZeneca, Lilly, Incyte, Bristol Myers Squibb (BMS), Agilent, Merck Serono, Sanofi, Pierre Fabre, GlaxoSmithKline (GSK), Novartis, Amgen and Janssen; and receiving support for attending meetings and/or travel from Astellas and Dako. VA reports personal honoraria from GSK, Pierre‐Fabre, Incyte. JG received honoraria for speaking by Roche Diagnostics. CL reports honoraria from Astellas (consulting and advisory board), and from Bayer, Gilead Sciences, MSD and Medica s.r.l. (consulting and speaker bureau). FB reports participation in an advisory board for AAA Novartis, Teysuno, Takeda; personal honoraria as invited speaker from Eli‐Lilly, MSD, Bayer, BMS, Pierre‐Fabre, AstraZeneca, Merck. SL reports personal honoraria as invited speaker from Amgen, Astra Zeneca, Bristol‐Myers Squibb, Incyte, GSK, Lilly, Merck Serono, MSD, Pierre‐Fabre, Roche, Servier. Participation in advisory board for Amgen, Astellas, Astra Zeneca, Bayer, BeOne, Bristol‐Myers Squibb, Daiichi‐Sankyo, Fosum Pharma, Gilead, GSK, Incyte, Lilly, Merck Serono, MSD, Nimbus Therapeutics, Rottapharm, Servier, Takeda.

## Supporting information


**Figure S1.** Comparison of the molecular landscape between the investigated adenosquamous carcinoma cohort and a gallbladder adenocarcinoma cohort. Data obtained from the sequencing of the GBAS cohort were compared with gallbladder adenocarcinomas available through cBioPortal (https://www.cbioportal.org/datasets), using ‘gallbladder’ as a filter condition. Three datasets are currently available (a) Gallbladder Cancer (MSK, 2022), (b) Gallbladder Cancer (MSK, Cancer 2018) and (c) Gallbladder Carcinoma (Shanghai, Nat Genet 2014). After the analysis of the clinical data, (b) and (c) were excluded, as in the (b) cohort, the histological features of the included cases were not clarified, while (c) cohort was composed only of Asiatic patients. The Gallbladder Cancer (MSK, 2022) dataset was selected to perform the comparison with the investigated cohort. The dataset comprised 208 gallbladder adenocarcinomas, investigated using the MSK‐IMPACT hybridization‐capture‐based NGS assay for targeted sequencing (doi: 10.1158/1078‐0432.CCR‐22‐1954). To reproduce similar experimental conditions, only specimens obtained from the primary tumour were selected. Moreover, patients were selected for their ethnicity, and Asian and black/Afro‐American patients were excluded. Finally, 63 patients of the Gallbladder Cancer (MSK, 2022) cohort were considered for the comparison with the investigated GBAS cohort. **p <0.01, ^1^data taken from doi: 10.1038/s41698‐022‐00327‐y. GBAC, gallbladder adenocarcinomas; TPS, tumour proportion score.
**Table S1.** Complete list of investigated genes and covered regions. *Exon that is only partially covered for SNV and indels.
**Table S2.** Molecular and immunohistochemical features of Claudin‐18‐stained cases. *For six cases, data about lymph node status were not available.
**Table S3.** Correlation between molecular, histological and immunohistochemical features. *One case is not adequate for the evaluation. **For six samples, the lymph node status was unavailable; percentages were calculated on 19 cases.

## Data Availability

The data that support the findings of this study are available from the corresponding author upon reasonable request.
