# Supplementary material for: Unveiling the molecular profile of adenosquamous gallbladder carcinoma: characterization of a Caucasian cohort
Source: Histopathology. 2026 May 29;89(3):442–55. doi: 10.1111/his.70179 (PMC13441353; doi:10.1111/his.70179)
Supplement: Supplementary file 1 [file HIS-89-442-s001.docx]

***Unveiling the molecular profile of adenosquamous gallbladder carcinoma: characterization of a Caucasian cohort***

Jessica Gasparello^1^, Valentina Angerilli^2,3,^ Mario Domenico Rizzato^4^, Edoardo Maffii^2^, Carlotta Ceccon^2^, Matilde Callegarin^1^, Marianna Sabbadin^2^, Monia Niero^2^, Luisa Toffolatti^2^, Francesca Bergamo^4^, Paola Parente^5^, Luca Mastracci^6,7^, Federica Grillo^6,7^, Alessandro Vanoli^8^, Paola Mattiolo^9^, Claudio Luchini^9^, Angelo Paolo Dei Tos^1^, Umberto Cillo^10,11^, Giacomo Zanus^10,12^, Sara Lonardi^4^, Matteo Fassan^1,4^

^1^Department of Medicine, University of Padua, Italy

^2^ Surgical Pathology Unit, ULSS2 Marca Trevigiana, Treviso, Italy

^3^Department of Pathology, Radboud Institute for Molecular Life Sciences, Radboud University Medical Centre, Nijmegen, The Netherlands

^4^Veneto Institute of Oncology, IOV-IRCCS, Padua, Italy

^5^Pathology Unit, Azienda Ospedaliero-Universitaria Policlinico Riuniti, Foggia, Italy

^6^Pathology Unit, Department of Surgical Sciences and Integrated Diagnostics (DISC), University of Genoa, Genoa, Italy

^7^IRCCS Azienda Ospedaliera Metropolitana (IRCCS AOM), Ospedale Policlinico San Martino, Genoa, Italy

^8^Anatomic Pathology Unit, IRCCS San Matteo Hospital Foundation, Pavia, Italy

^9^Department of Pathology and Diagnostics, University and Hospital Trust of Verona, Verona, Italy

^10^Department of Surgical, Oncological and Gastroenterological Sciences (DISCOG), University of Padua, Italy

^11^Department of Surgery, General Surgery 2-HPB Surgery and Liver Transplantation Unit, Padua University Hospital, Italy

^12^General Surgery 2, ULSS2 Marca Trevigiana, Italy

**Correspondence to**: Matteo Fassan; Department of Medicine (DIMED); Surgical Pathology Unit; University of Padua; via Gabelli 61, 35121 Padua - ITALY; Phone: (+39) 049 8211312; e-mail: matteo.fassan@unipd.it

**Running title:** Molecular characterization of adenosquamous gallbladder carcinoma cohort

**Keywords:** adenosquamous gallbladder carcinoma, gallbladder cancer, molecular profile, next-generation sequencing, targeted therapy.

**Supplementary materials**

**Supplementary Figure 1. Comparison of the molecular landscape between the investigated adenosquamous carcinoma cohort and a gallbladder adenocarcinoma cohort.** Data obtained from the sequencing of the GBAS cohort were compared with gallbladder adenocarcinomas available through cBioPortal (https://www.cbioportal.org/datasets), using ‘gallbladder’ as a filter condition. Three datasets are currently available (a) Gallbladder Cancer (MSK, 2022), (b) Gallbladder Cancer (MSK, Cancer 2018), (c) Gallbladder Carcinoma (Shanghai, Nat Genet 2014). After the analysis of the clinical data, (b) and (c) were excluded, as in the (b) cohort, the histological features of the included cases were not clarified, while (c) cohort was composed only of Asiatic patients. The Gallbladder Cancer (MSK, 2022) dataset was selected to perform the comparison with the investigated cohort. The dataset comprised 208 gallbladder adenocarcinomas, investigated using the MSK-IMPACT hybridization-capture-based NGS assay for targeted sequencing (doi: 10.1158/1078-0432.CCR-22-1954). To reproduce similar experimental conditions, only specimens obtained from the primary tumor were selected. Moreover, patients were selected for their ethnicity, and Asian and black/afro-american patients were excluded. Finally, 63 patients of the Gallbladder Cancer (MSK, 2022) cohort were considered for the comparison with the investigated GBAS cohort. ** p<0.01, ^1^data taken from doi.10.1038/s41698-022-00327-y, TPS: tumor proportion score, GBAC: gallbladder adenocarcinomas.

| Gene | Accession number | Covered exons | Detected alterations | |
| --- | --- | --- | --- | --- |
|  |  |  | SNV/indel | CNV |
| *ABL1* | NM_005157 | 4,5,6,7 | yes | yes |
| *AKT1* | NM_005163 | 3,6 | yes | no |
| *ALK* | NM_004304 | 21,22,23,25 | yes | yes |
| *APC* | NM_000038 | 16 | yes | yes |
| *ATM* | NM_000051 | 8,9,12,17,26,34,35,36,39,50,54,55,56,59,61,63 | yes | yes |
| *AURKA* | NM_003600 | 2,5,6,7,8 | yes | yes |
| *BRAF* | NM_004333 | 11,15 | yes | no |
| *CCND1* | NM_053056 | na | no | yes |
| *CCNE1* | NM_001238 | 3,4,5,6,7,8,10,12 | yes | yes |
| *CDH1* | NM_004360 | 1,2,3,4,5,6,7,8,9,10,11,12,13,14,15,16 | yes | yes |
| *CDK4* | NM_000075 | 2,3,4,5,6,7 | yes | yes |
| *CDKN2A* | NM_000077 | 1,2,3 | yes | yes |
| *CSF1R* | NM_005211 | 7,22 | yes | no |
| *CTNNB1* | NM_001904 | 3 | yes | no |
| *DDR2* | NM_006182 | 12,13,14,15,16,17,18 | yes | yes |
| *EGFR* | NM_005228 | 3,7,15,18,19,20,21 | yes | yes |
| *ERBB2* | NM_004448 | 10,19,20,21,24 | yes | yes |
| *ERBB3* | NM_001982 | 2,3,7,8 | yes | yes |
| *ERBB4* | NM_005235 | 3,4,6,7,8,9,15,23 | yes | yes |
| *ESR1* | NM_000125 | 8 | yes | no |
| *EZH2* | NM_004456 | 16 | yes | no |
| *FBXW7* | NM_018315 | 1,2,3,4,5,6,7,8,9,10,11 | yes | yes |
| *FGFR1* | NM_015850 | 4,7,8,13,15,17 | yes | yes |
| *FGFR2* | NM_000141 | 7,9,12,14 | yes | yes |
| *FGFR3* | NM_000142 | 7,8,9,14,15,16,18 | yes | yes |
| *FLT3* | NM_004119 | 11,14,16,20 | yes | yes |
| *FOXL2* | NM_023067 | 1 | yes | no |
| *GNA11* | NM_002067 | 5 | yes | no |
| *GNAQ* | NM_002072 | 4,5 | yes | no |
| *GNAS* | NM_000516 | 6,7,8,9 | yes | yes |
| *H3F3A* | NM_002107 | 2 | yes | no |
| *HNF1A* | NM_000545 | 3.4 | yes | no |
| *HRAS* | NM_005343 | 2,3 | yes | no |
| *IDH1* | NM_005896 | 3,4 | yes | no |
| *IDH2* | NM_002168 | 4 | yes | no |
| *JAK2* | NM_004972 | 11,13,14,16,19 | yes | yes |
| *JAK3* | NM_000215 | 4,13,16 | yes | yes |
| *KDR* | NM_002253 | 6,7,11,19,21,26,27,30 | yes | yes |
| *KIT* | NM_000222 | 2,8,9,10,11,13,14,15,17,18 | yes | yes |
| *KRAS* | NM_004985 | 2,3,4,5 | yes | yes |
| *MAP2K1* | NM_002755 | 2,3 | yes | no |
| *MDM2* | NM_002392 | 2,3,4,6,8*10 | no | yes |
| *MET* | NM_000245 | 2,11,14,16,19,21 | yes | yes |
| *MYC* | NM_002467 | na | no | yes |
| *MYCN* | NM_005378 | na | no | yes |
| *MLH1* | NM_000249 | 12 | yes | no |
| *MPL* | NM_005373 | 10 | yes | no |
| *NOTCH1* | NM_017617 | 25,26,27,34 | yes | yes |
| *NPM1* | NM_002520 | 11 | yes | no |
| *NRAS* | NM_002524 | 2,3,4,5 | yes | yes |
| *PDGFRA* | NM_006206 | 12,14,15,18,23 | yes | yes |
| *PIK3CA* | NM_006218 | 2,5,7,8,10,14,19,21 | yes | yes |
| *PIK3R1* | NM_181504 | 1,2,3,4,5,6,7,8,9,10 | yes | yes |
| *PTEN* | NM_000314 | 1,2,3,4,5,6,7,8,9 | yes | yes |
| *PTPN11* | NM_002834 | 3,13 | yes | no |
| *RB1* | NM_000321 | 4,6,10,11,14,17,18,20,21,22* | yes | yes |
| *RET* | NM_020630 | 10,11,13,14,15,16 | yes | yes |
| *RHOA* | NM_001664 | 2,3 | yes | no |
| *ROS1* | NM_002944 | 38 | yes | no |
| *SMAD4* | NM_005359 | 2,3,4,5,6,7,8,9,10,11,12 | yes | yes |
| *SMARCB1* | NM_003073 | 2,4,5,9 | yes | yes |
| *SMO* | NM_005631 | 3,5,6,9,11 | yes | yes |
| *SRC* | NM_005417 | 14 | yes | no |
| *STK11* | NM_000455 | 1,2,3,4,5,6,7,8,9 | yes | yes |
| *TERT* | NM_198253 | Promoter, 1 | yes | no |
| *TP53* | NM_000546 | 1,2,3,4,5,6,7,8,9,10,11 | yes | yes |
| *VHL* | NM_000551 | 1,2,3 | yes | yes |

**Supplementary Table 1. Complete list of investigated genes and covered regions.** *exon that is only partially covered for SNV and indels.

| Features | | CLDN-18 positive | CLDN-18 negative | p-value |
| --- | --- | --- | --- | --- |
| Sex | Male (12) | 3/12 (25%) | 9/12 (75%) | *p=0.7482* |
|  | Female (13) | 4/13 (30.8%) | 9/13 (69.2%) |  |
| Age at diagnosis | <65 (8) | 3/8 (37.5%) | 5/8 (62.5%) | *p=0.4680* |
|  | >65 (17) | 4/17 (23.5%) | 13/17 (76.5%) |  |
| pT | T1-T2 (2) | 1/2 (50%) | 1/2 (50%) | *p=0.4700* |
|  | T3-T4 (23) | 6/23 (26.1%) | 17/23 (73.9%) |  |
| pN* | N0 (10) | 4/10 (40%) | 6/10 (60%) | *p=0.1533* |
|  | N1-N2 (9) | 1/9 (11.1%) | 8/9 (88.9%) |  |
| pM | M0 (18) | 3/18 (16.7%) | 15/18 (83.3%) | *p=0.0430* |
|  | M1 (7) | 4/7 (57.1%) | 3/7 (42.9%) |  |
| Squamous differentiation (%) | <75 (14) | 6/14 (42.9%) | 8/14 (57.1%) | *p=0.090* |
|  | ≥75 (11) | 1/11 (9.1%) | 10/11 (90.9%) |  |
| Intestinal  metaplasia | Yes (5) | 2/5 (40%) | 3/5 (60%) | *p=0.5040* |
|  | No (20) | 5/20 (25%) | 15/20 (75%) |  |
| *TP53* status | Mutated (16) | 2/16 (12.5%) | 14/16 (87.5%) | *p=0.0214* |
|  | Wild type (9) | 5/9 (55.6%) | 4/9 (44.4%) |  |
| *PIK3CA* status | Mutated (10) | 4/10 (40%) | 6/10 (60%) | *p=0.2752* |
|  | Wild type (15) | 3/15 (20%) | 12/15 (80%) |  |
| *PTEN* status | Mutated (6) | 1/6 (16.7%) | 5/6 (83.3%) | *p=0.4782* |
|  | Wild type (19) | 6/19 (31.6%) | 13/19 (68.4%) |  |
| *SMAD4* status | Mutated (6) | 2/6 (33.3%) | 4/6 (66.7%) | *p=0.7386* |
|  | Wild type (19) | 5/19 (26.3%) | 14/19 (73.7%) |  |
| *N° of mutations* | <3 (11) | 3/11 (27.3%) | 8/11 (72.7%) | *p=0.4604* |
|  | 3-6 (11) | 4/11 (36.4%) | 7/11 (63.6%) |  |
|  | >6 (3) | 0/3 (0%) | 3/3 (100%) |  |

**Supplementary Table 2. Molecular and immunohistochemical features of Claudin-18-stained cases.** *For six cases, data about lymph node status were not available.

|  |  | ***TP53* status** | | | ***PIK3CA* status** | | | ***PTEN* status** | | | **N° of mutations** | | | |
| --- | --- | --- | --- | --- | --- | --- | --- | --- | --- | --- | --- | --- | --- | --- |
|  |  | Mutated (16/25) | Wild type (9/25) | p-value | Mutated (10/25) | Wild type  (15/25) | p-value | Mutated (6/25) | Wild type (19/25) | p-value | < 3 (11/25) | 3-6 (11/25) | > 6 (3/25) | p-value |
| Age at diagnosis | <65 | 7/16 (43.8%) | 1/9 (11.1%) | 0.0931 | 5/10 (50%) | 3/15 (20%) | 0.1152 | 3/6 (50%) | 5/19 (26.3%) | 0.2783 | 3/11 (27.3%) | 4/11 (36.4%) | 1/3 (33.3%) | 0.8996 |
|  | >65 | 9/16 (56.2%) | 8/9 (88.9%) |  | 5/10 (50%) | 12/15 (80%) |  | 3/6 (50%) | 14/19 (73.7%) |  | 8/11  (72.7%) | 7/11 (63.6%) | 2/3  (66.7%) |  |
| PD-L1 status* (CPS) | <10 | 5/16 (31.2%) | 4/9 (44.4%) | 0.3711 | 5/10 (50%) | 4/15 (26.7%) | 0.2850 | 1/6 (16.7%) | 6/19 (31.6%) | 0.4367 | 5/11 (50%) | 2/11 (18.2%) | 0/3 (0%) | 0.1368 |
|  | ≥10 | 11/16 (68.8%) | 4/9 (44.4%) |  | 5/10 (50%) | 10/15 (66.7%) |  | 5/6 (83.3%) | 12/19 (63.2%) |  | 5/11 (50%) | 9/11 (81.8%) | 3/3 (100%) |  |
| pT | T1-T2 | 1/16 (6.2%) | 1/9 (11.1%) | 0.6672 | 2/10 (20%) | 0/15 (0%) | 0.0710 | 1/6 (16.7%) | 1/19 (5.3%) | 0.3694 | 1/11 (9.1%) | 1/11 (9.1%) | 0/4 (0%) | 0.8622 |
|  | T3-T4 | 15/16 (93.8%) | 8/9 (88.9%) |  | 8/10 (80%) | 15/15 (100%) |  | 5/6 (83.3%) | 18/19 (94.7%) |  | 10/11 (90.9%) | 10/11 (90.9%) | 3/3 (100%) |  |
| pN** | N0 | 6/12 (50%) | 4/7 (57.1%) | 0.7636 | 3/5 (60%) | 7/14 (50%) | 0.7007 | 1/2 (50%) | 9/17 (52.9%) | 0.4217 | 7/11 (63.6%) | 2/6 (33.3%) | 1/2  (50%) | 0.4877 |
|  | N1-2 | 6/12  (50%) | 3/7 (42.9%) |  | 2/5 (40%) | 7/14 (50%) |  | 1/2 (50%) | 8/17 (47.1%) |  | 4/11 (36.4%) | 4/6 (66.6%) | 1/2  (50%) |  |
| pM | M0 | 13/16 (81.3%) | 5/9 (55.6%) | 0.1696 | 8/10 (80%) | 10/15 (66.7%) | 0.4670 | 6/6 (100%) | 12/19 (63.2%) | 0.0797 | 8/11 (72.7%) | 7/11 (63.6%) | 3/3 (100%) | 0.4604 |
|  | M1 | 3/16 (18.7%) | 4/9 (44.4%) |  | 2/10 (20%) | 5/15 (33.3%) |  | 0/6 (0%) | 7/19 (36.8%) |  | 3/11 (27.3%) | 4/11 (36.4%) | 0/3 (0%) |  |
| % of squamous differentiation | <75% | 6/16 (37.5%) | 8/9 (88.9%) | 0.4760 | 7/10 (70%) | 11/15 (73.3%) | 0.8557 | 2/6 (33.3%) | 12/19 (63.2%) | 0.1995 | 9/11 (81.8%) | 6/11 (54.5%) | 0/3 (0%) | 0.0331 |
|  | >75% | 10/16 (62.5%) | 1/9 (11.1%) |  | 3/10 (30%) | 4/15 (26.7%) |  | 4/6 (66.7%) | 7/19 (36.8%) |  | 2/11 (18.2%) | 5/11 (45.5%) | 3/3 (100%) |  |
|  | Mean | 66.6 | 50.6 |  | 69 | 55.3 |  | 68.3 | 58.4 |  | 49.5 | 67.2 | 78.3 |  |
|  | Median | 77.5 | 50 |  | 80 | 50 |  | 80 | 50 |  | 50 | 75 | 80 |  |
| BilIN | Yes | 15/16 (93.7%) | 5/9 (55.6%) | 0.0219 | 9/10 (90%) | 11/15 (73.3%) | 0.3074 | 6/6 (100%) | 14/19 (73.7%) | 0.1601 | 9/11 (81.8%) | 9/11 (81.8%) | 2/3 (66.7%) | 0.8275 |
|  | No | 1/16 (6.3%) | 4/9 (44.4%) |  | 1/10 (10%) | 4/15 (26.7%) |  | 0/6 (0%) | 5/19 (26.3%) |  | 2/11 (18.2%) | 2/11 (18.2%) | 1/3 (33.3%) |  |
| Squamous  component | Intermingled | 11/16  (68.8%) | 3/9  (33.3%) | 0.1153 | 4/10  (40%) | 10/15  (66.7%) | 0.1882 | 4/6  (66.7%) | 10/19  (52.6%) | 0.5460 | 6/11  (54.5%) | 6/11  (54.5%) | 2/3  (66.7%) | 0.9242 |
|  | Distinct | 5/16  (31.2%) | 6/9  (66.7%) |  | 6/10  (60%) | 5/15  (33.3%) |  | 2/6  (33.3%) | 9/19  (47.4%) |  | 5/11  (45.5%) | 5/11  (54.5%) | 1/3  (33.3%) |  |

**Supplementary Table 3. Correlation between molecular, histological, and immunohistochemical features.** *One case is not adequate for the evaluation. ** For 6 samples, the lymph node status was unavailable; percentages were calculated on 19 cases.
